# Thymidine Kinase^+/−^ Mammalian Cell Mutagenicity Assays for Assessment of Nanomaterials

**DOI:** 10.3389/ftox.2022.864753

**Published:** 2022-06-08

**Authors:** Tao Chen, Maria Dusinska, Rosalie Elespuru

**Affiliations:** ^1^ Division of Genetic and Molecular Toxicology, National Center for Toxicological Research, U.S. Food and Drug Administration, Jefferson, AR, United States; ^2^ Health Effects Laboratory, NILU-Norwegian Institute for Air Research, Kjeller, Norway; ^3^ Division of Biology, Chemistry and Materials Science, US Food and Drug Administration, CDRH/OSEL, Silver Spring, MD, United States

**Keywords:** nanomaterials, mouse lymphoma, TK6, mammalian mutagenicity, mutagenesis

## Abstract

The methods outlined here are part of a series of papers designed specifically for genotoxicity assessment of nanomaterials (NM). Common Considerations such as NM characterization, sample preparation and dose selection, relevant to all genotoxicity assays, are found in an accompanying paper. The present paper describes methods for evaluation of mutagenicity in the mammalian (mouse) *thymidine kinase* (*Tk*) gene occurring in L5178Y mouse lymphoma (ML) cells and in the designated *TK* gene in human lymphoblastoid TK6 cells. Mutations change the functional genotype from TK^+/−^ to TK^−/−^, detectable as cells surviving on media selective for the lack of thymidine kinase (TK) function. Unlike cells with TK enzyme function, the TK^−/−^ cells are unable to integrate the toxic selection agent, allowing these cells to survive as rare mutant colonies. The ML assay has been shown to detect a broad spectrum of genetic damage, including both small scale (point) mutations and chromosomal alterations. This assay is a widely used mammalian cell gene mutation assay for regulatory purposes and is included in the core battery of genotoxicity tests for regulatory decision-making. The TK6 assay is an assay using a human cell line derived similarly via mutagenic manipulations and optimal selection. Details are provided on the materials required, cell culture methods, selection of test chemical concentrations, cytotoxicity, treatment time, mutation expression, cloning, and data calculation and interpretation. The methods describe the microwell plate version of the assays without metabolic activation.

## Graphical Abstract

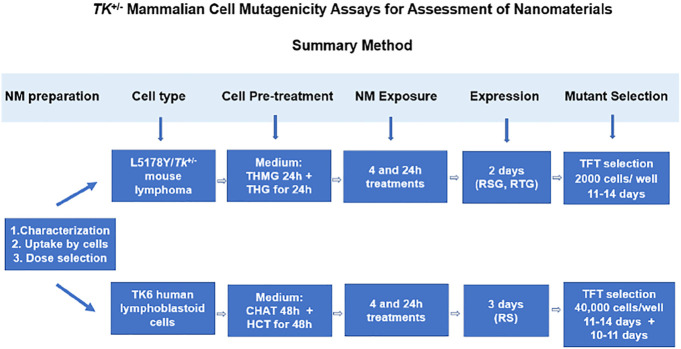

## 1 Introduction

The methods found in the Nanotechnology Section of *Frontiers in Toxicology* are a follow-up to the analysis and critique of the literature on genotoxicity assessment of nanomaterials (NM) by an international group working together via the Genetic Toxicology Testing Committees (GTTC) of the Health and Environmental Sciences Institute (Elespuru et al., 2018). The mammalian TK mutagenicity assays described here use the *TK (thymidine kinase)* gene as a target for mutational analysis. OECD Test Guideline (TG) 490 describes parameters for performing the assays (OECD 490, 2016), but the TG does not address methods specific to valid assessment of NM.

The Mouse Lymphoma Assay (MLA), using the heterozygous rodent L5178Y mouse lymphoma cells, is widely used in the genotoxicity test battery because of its capability of detecting both small-scale mutational damage and large-scale chromosomal alterations. Large portions of the gene may be lost without compromising viability, thus allowing the detection of a broad spectrum of genetic alterations (Clive and Spector, 1975; Applegate et al., 1990). The human lymphoblastoid TK6 cells (Skopek et al., 1978; Liber and Thilly, 1982) were developed in the same time frame as the MLA and are considered useful because of the human origin of the cells, which are P53 proficient. However, these cells originated from a person with a hereditary genetic disease and were manipulated by mutagen treatment and selection for their growth capability (Skopek et al., 1978). These cells have come into general use relatively recently. The two cell types are functionally similar as targets for mutation induction and useful as laboratory models for mammalian mutagenesis. (Note that genetic nomenclature is different for human and rodent cells; thus, the differences used here: *Tk* for the mouse gene, *Tk* for the human gene, *T_k_* for the thymidine kinase protein, and *TK* generally referring to genes in both assays).

The assays detect a broad spectrum of genetic damage due to the nature and autosomal location of the TK gene. Two distinct phenotypic classes of TK mutants are generated in these assays, the normal growing and slow growing mutants that are recognized as large colony and small colony mutants, respectively, in the MLA. They are early appearing and late appearing colony mutants in the TK6 assay. Slow growing mutants of both cell types have acquired genetic damage that involves putative growth-regulating gene(s) near the TK locus, resulting in prolonged doubling times and the formation of late appearing or small colonies. More recent studies have demonstrated the molecular nature of mutations in both assays (Hakulinen et al., 2011; Guo et al., 2018). Other results indicate that the assays are sensitive enough to detect mutagenicity of NM (Mei et al., 2012; Elespuru et al., 2018; Demir et al., 2020).

The methods presented in this paper are written specifically for mutagenicity evaluation of NM, providing details of the materials required, cell culture methods, selection of test chemical concentrations, cytotoxicity, treatment times, mutation fixing, cloning, and data calculation and interpretation. The detailed protocols describe the microwell plate version of the assays without metabolic activation. The methods are meant to be used together with the accompanying paper on Common Considerations for Genotoxicity Assessment of Nanomaterials (*Front. Toxicol. doi: 10.3389/ftox.2022.859122*). NM issues related specifically to this assay include dosimetry (toxicity, agglomeration), separation of the NM from the suspension cells, and effects on mutant expression that may require extended incubation times.

## 2 Test Systems

### 2.1 Cells

Cell types: L5178Y/*Tk* ^+/−^ −3.7.2°C subline, derived from a mouse lymphoma (Clive and Spector, 1975), and TK6, a lymphoblastoid cell line derived from a human genetic disease source (Skopek et al., 1978). Both cell types are grown in suspension culture and maintained in exponential growth for the assay. See (Lorge et al., 2016) for additional information on methods for handling cells.

### 2.2 Media

• The basic medium (F_0P_) consists of RPMI 1640 medium supplemented with 100 unit/mL penicillin, 100 mg/ml streptomycin, and 200 mg/ml sodium pyruvate.
• Treatment medium (F_5P_) contains 5% (v/v) heat-inactivated horse serum or heat-inactivated fetal calf serum in F_0P_. Note that if the serum is not purchased as heat inactivated serum, it requires heat-inactivation at 56°C for 30 min before use.
• Growth medium (F_10P_) contains 10% (v/v) serum added to F_0P_.
• Cloning medium (F_20P_) contains 20% (v/v) serum added to F_0P_.
• THMG stock medium (100x) consists of F_0P_ supplemented with 300 mg/ml thymidine, 500 mg/ml hypoxanthine, 10 mg/ml methotrexate and 750 mg/ml glycine.
• THG stock medium (100x) contains the same components as THMG stock medium without methotrexate.
• CHAT medium contains F_10P_ supplemented with 10 μM 2′‐deoxycytidine, 200 μM hypoxanthine, 0.1 μM aminopterin, and 17.5 μM thymidine.
• HCT medium (CHAT without aminopterin)
• TFT mutant selection medium contains 100 μg/ml trifluorothymidine in saline

Media are filter-sterilized and stored at 4°C wrapped in foil to protect from light. THMG, THG, CHAT and HCT media can be stored at −20°C. The media are warmed to room temperature before use.

### 2.3 Maintenance of Cells

The cultures are grown in polycarbonate tissue culture flasks and placed in a 95% humidified incubator with 5% CO_2_-in- air at 37°C. For a valid assay the cells need to be maintained in log phase; doubling times are 9–10 h for L5178Y and 11–12 h for TK6. The cultures are routinely diluted with fresh F_10P_ medium each day to 2 × 10^5^ cells/mL. For longer periods, the cells can be diluted to 7 × 10^3^ cells/mL Doubling times must be carefully monitored because cultures showing prolonged doubling times should not be used for experiments. Cells can be cryopreserved in liquid N_2_ using F_20P_ containing 5% dimethyl sulfoxide (DMSO).

### 2.4 Cleansing Cell Cultures

An essential requirement is elimination of pre-existing *TK* ^*−/−*^ cells that would impact the mutation levels in the negative controls in the test. Cleansing is carried out during the week preceding an assay or prior to freezing many vials for storage in liquid N_2_. Note that the cells grow at longer doubling times during cleansing. The cells should not be exposed to test chemicals until they have completely recovered from cleansing, usually after 24 h. Cleansed cells can be cryopreserved at a density of 5 × 10^6^ cells/mL/tube in freezing medium (e.g., 10% DMSO in cell culture medium with serum). New cultures for assays may be started directly from the cryopreserved cleansed stocks, after centrifuging and re-suspended in fresh medium.

To cleanse L5178Y: Cells are treated sequentially with THMG and then THG, as follows: 0.5 ml of THMG (100X stock) is added to 50 ml of cell culture at 2 × 10^5^ cells/mL in F_10P_. The cells are incubated at 37°C for 24 h in this medium. After counting (the cell density should be ∼1.0 × 10^6^ cells/mL), cells are centrifuged at 200 x *g* for 10 min and the pellet is resuspended at a concentration of 2 × 10^5^ cells/mL in 1% THG medium (F_10P_ medium containing 1% THG stock) and incubated for another 24 h.

To cleanse TK6: Cells are incubated in CHAT medium for 48 h and then transferred into HCT medium for 48 h to kill *TK* ^*−/−*^ cells before starting the *TK* assay.

## 3 Characterization of NM

Refer to the Common Considerations paper for this information [*Front. Toxicol. doi: 10.3389/ftox.2022.859122*].

## 4 Preparation of NM for Testing

Most NM are not soluble in aqueous solutions. Make sure solutions are made fresh right before the experiment. A test NM stock solution is prepared by dispersion of the NM in sterilized deionized H_2_O or another suitable aqueous solvent by vortexing and then sonicating to ensure a uniform suspension of the NM. Then the stock solutions can be diluted to different concentrations with treatment medium. Usually, NM are not sterilized prior to use because of potential alterations to the material, but all other techniques should be performed under sterile conditions.

## 5 Preliminary Experiments

### 5.1 Positive and Negative Controls

To ensure that an assay is valid, positive controls and negative controls are included in each experiment. Chemicals commonly used as positive controls for test of any agent in TK mutagenicity assays include methylmethanesulphonate (MMS) at 10–20 mg/ml and 4-nitroquinoline 1-oxide (NQO) at 0.05–0.1 mg/ml. NQO can be prepared in DMSO as a 100-fold concentrated stock solution and stored as frozen aliquots at −80°C ***.*** However, MMS 100X stocks should be freshly prepared with physiological saline. Reagent solutions should be protected from light. If a solvent other than saline or F_0P_ (culture medium minus serum) is used, the negative control should receive a volume of the solvent equivalent to the highest amount used for a treated culture without exceeding a final volume of 1%. A NM-type positive control, such as Tungsten carbide-cobalt (WC-Co), would be useful; however, validation studies have not been carried out, and widespread use of this or other potential NM positive controls have not occurred. Consensus has not been found yet for a positive NM control for this (or any other genotoxicity) assay. The general requirement that a positive control generate a strong response may prevent the use of WC-Co and other NM as positive controls, since their responses generally are relatively weak. Thus, genotoxicity assessment of NM is carried out using common positive controls for each assay.

### 5.2 Determination of Exposure Concentrations

If the cytotoxicity of the test NM is unknown in this test system, a preliminary experiment should be performed to define the cytotoxic concentration range. The test NM is suspended in a suitable solvent such as saline, DMSO, or F_0P_ (cell culture medium with no serum) at appropriate concentrations of stock solutions. Stock solutions are created that can be diluted into the test system to generate the desired concentration. Sets of stock solutions are prepared so that the same dilution into the test system (e.g., 1:10 or 1:100) is made for each concentration. If possible, aqueous vehicles compatible with the cell systems should be chosen. Appropriate amounts of stock solutions are added to the cells in suspension in treatment medium to create the desired final concentrations for each data point in the experiment. The volume added should not exceed 1% (i.e., at least 1:100 dilution) when DMSO or other non-aqueous solvents are used. Such dilutions are necessary to avoid toxic effects of non-aqueous solvents on the test cells. The final volume of solvent or vehicle should be the same in all cultures.

The exposure concentrations of a test NM are selected according to target toxicity ranges, with a maximum allowable cytotoxicity of 10% growth relative to the negative control [see (OECD 490, 2016)]. The exposure concentration selected is also dependent on agglomeration status of the test article, which may signal an upper limit of exposure (see the Common Considerations paper). Generally, one or more preliminary experiments using half-log dilutions may be useful to aid in determining the concentrations used in the definitive assay. Duplicate (or triplicate) cultures are used for the negative/solvent control. See the Common Considerations paper for additional information on selection of concentrations for study (*Front. Toxicol. doi: 10.3389/ftox.2022.859122*)*.*

## 6 Definitive Assays

### 6.1 Chemical Treatment

Selection of concentrations for test may have been informed by a preliminary experiment determining reasonable limits of high and low concentrations selected for assessment. In that case, 4-5 useful concentrations with a relatively narrow concentration range (2 or 3-fold) between exposures may be selected. If a preliminary concentration-response assessment has not been made, then additional test concentrations should be used as a better likelihood of finding an informative dose range. Test concentrations ideally range between non-toxic and moderately toxic (90% survival loss). With many NM it may not be feasible to test into a moderately toxic zone, due to agglomeration or other effects.

The cells should always be maintained in logarithmic growth before cell treatment. For treatment, the test NM solution or suspension, positive control chemicals, or control vehicle are added into 50-ml sterile disposable centrifuge tubes containing 6 × 10^6^ cells for L5178Y or 10^6^ cells for TK6 in 10 ml of F_5P_ (cell culture medium with 5% serum). After gentle mixing, cultures are placed in a CO_2_ incubator at 37°C for 4 h. A 24 h treatment should be conducted in parallel, or subsequently if the 4 h treatment is negative. For the 24 h treatment, the cell density should be adjusted to 2 × 10^5^ cells/mL to allow for additional growth over the longer incubation period. Cultures may be placed on a rocker platform during treatment to prevent the NM from settling out. After incubation, the cells are centrifuged at 200 x *g* for 10 min and the supernatant is discarded. Each culture is then washed twice with F_0P_, to remove the NM test article, by centrifuging and resuspending the cells in fresh medium. Notation should be made of visible remaining NM, and a third wash instituted if necessary. After the final centrifugation, the cell pellet is resuspended in 20 ml of fresh F_10P_ (cell culture medium with 10% serum) at a concentration of 3 × 10^5^ cells/mL.

### 6.2 Expression Growth

After treatment, the cells are cultured for an expression period of 48 h for L5178Y or 72 h for TK6 for DNA damage processing and mutation fixation. Cell densities are measured approximately 24 h following treatment and adjusted to 2 × 10^5^ cells/mL with fresh F_10P_. On completion of the 48- or 72-h expression period, cell densities are measured again. The cell densities from each expression day are used in calculating the relative suspension growth (RSG) and the relative total growth (RTG) for L5178Y or the RS for TK6 [see (OECD 490, 2016)]. Cultures with cell densities less than 2 × 10^5^/ml are not used for cloning and mutagenicity measurements.

### 6.3 Cloning (Mutant Selection)

Trifluorothymidine (TFT) stock solution is made with 10 mg TFT in 100 ml physiological saline and stored in a foil-wrapped bottle. The stock solution is filter sterilized and can be dispensed in 15-ml aliquots in sterile tubes and stored at −20°C for up to 3 months.

For cloning, each culture is centrifuged, and the cell pellet resuspended in F_20P_ (cell culture medium with 20% serum) at a density of 2 × 10^5^ cells/mL. The cells should be single cell suspensions, so that individual cells are plated and the colonies that form are from single cells. The cultures are incubated for 30 min to minimize trauma and allow them to adapt to the medium. The cells are then diluted to the appropriate densities to plate for TFT resistance and cell viability.

For the TFT resistant plating (mutant colonies), the cell concentrations are adjusted to 1 × 10^4^/ml in F_20P_ for L5178Y and 2 × 10^5^/ml for TK6 cells. Then TFT (3 mg/ml) is added to the selection flask. Using a multichannel pipette, 200 ml of each TFT containing suspension is placed into each well of 4 flat-bottomed 96-well plates. For L5178 the final density is 2000 cells/well; for TK6 it is 40,000 cells/well. Colonies are identified by low power microscope or by visual observation. Small colonies are defined as less than a quarter of the diameter of the well while large colonies are more than a quarter of the diameter of the well. The morphology is generally compact for small colonies and may be diffuse for large colonies.

For the determination of plating efficiency, the cultures are adjusted to 8 cells/mL in media without TFT and 200 µL per well are aliquoted into two 96-well flat-bottom microtiter plates (∼1 or 2 cells/well) for the counting of survivors. The microtiter plates are incubated at 37°C in a humidified incubator with 5% CO2-in-air for 11–14 days for the L5178Y *Tk* mutants. An additional period of 10–14 days is required for the TK6 slower growing colonies to appear.

## 7 Data Analysis

### 7.1 Mutant Frequency (Mutant/Survivor)

The mutant frequency (MF) is determined by the plating efficiencies of mutant colonies (PE_M_) and adjusted with plating efficiencies of viable cells (PE_V_) from the same culture. See OECD 490 for details (OECD 490, 2016).

### 7.2 Assay Acceptibility

• The positive control must demonstrate the assay is properly conducted and that small or late growing colony mutants were detected. This is demonstrated by a significantly induced small colony mutant (MLA) or late growing colony (TK6) mutation frequency in the positive controls.
• RS/RTG >10%
• Spontaneous mutation frequency is 50–170 × 10^−6^ for MLA and ∼3.1 ± 1.1 × 10^−6^ for TK6
• Cloning Efficiency for negative control: 65–120% for MLA and >65% for TK6
• Suspension Growth for negative control (corrected for cytotoxicity during treatment and during expression): 8- to 32-fold for the MLA

### 7.3 Criteria for Positive and Negative Results

These criteria are found in OECD 490 and represent the work of several IWGT MLA Workgroups.

#### 7.3.1 For MLA

• A positive test chemical response requires an induced MF of at least 126 × 10^−6^ for the microwell version of the assay. This is termed the global evaluation factor (GEF). A compound is called negative if it does not meet the criteria for a positive response when the RTG reaches 10–20%.

#### 7.3.2 For TK6

• At least one of the test concentrations exhibits a statistically significant increase compared with the concurrent negative control.
• There is a dose-related trend
• Any of the results are outside the distribution of the historical negative control.

## 8 Summary

Mammalian TK mutagenicity assays are recommended for assessment of genetic interactions leading to mutations reflecting heritable sequence changes in the DNA. Two systems described here have been used in laboratories around the world for genotoxicity assessment of chemicals and agents. This paper provides a detailed protocol for use of one or the other of the assays in assessment of NM genotoxicity. NM are a diverse set of agents, often with unique chemical and physical properties that can impact the assays in ways both expected and unexpected. NM generally require special handling for valid assessment of NM, with attention to properties that affect their assessment, such as agglomeration and distribution within biological systems, as well as properties that impact the assays themselves. A detailed protocol is provided for assessment of NM mutagenicity.

## Data Availability

The original contributions presented in the study are included in the article/supplementary material, further inquiries can be directed to the corresponding author.
